# Profiling Murine Tau with 0N, 1N and 2N Isoform-Specific Antibodies in Brain and Peripheral Organs Reveals Distinct Subcellular Localization, with the 1N Isoform Being Enriched in the Nucleus 

**DOI:** 10.1371/journal.pone.0084849

**Published:** 2013-12-30

**Authors:** Chang Liu, Jürgen Götz

**Affiliations:** 1 Sydney Medical School, Brain and Mind Research Institute, University of Sydney, Camperdown, New South Wales, Australia; 2 Clem Jones Centre for Ageing Dementia Research (CJCADR), Queensland Brain Institute (QBI), The University of Queensland, St Lucia Campus, Brisbane, Queensland, Australia; New York State Institute for Basic Research, United States of America

## Abstract

In the adult murine brain, the microtubule-associated protein tau exists as three major isoforms, which have four microtubule-binding repeats (4R), with either no (0N), one (1N) or two (2N) amino-terminal inserts. The human brain expresses three additional isoforms with three microtubule-binding repeats (3R) each. However, little is known about the role of the amino-terminal inserts and how the 0N, 1N and 2N tau species differ. In order to investigate this, we generated a series of isoform-specific antibodies and performed a profiling by Western blotting and immunohistochemical analyses using wild-type mice in three age groups: two months, two weeks and postnatal day 0 (P0). This revealed that the brain is the only organ to express tau at significant levels, with 0N4R being the predominant isoform in the two month-old adult. Subcellular fractionation of the brain showed that the 1N isoform is over-represented in the soluble nuclear fraction. This is in agreement with the immunohistochemical analysis as the 1N isoform strongly localizes to the neuronal nucleus, although it is also found in cell bodies and dendrites, but not axons. The 0N isoform is mainly found in cell bodies and axons, whereas nuclei and dendrites are only slightly stained with the 0N antibody. The 2N isoform is highly expressed in axons and in cell bodies, with a detectable expression in dendrites and a very slight expression in nuclei. The 2N isoform that was undetectable at P0, in adult brain was mainly found localized to cell bodies and dendrites. Together these findings reveal significant differences between the three murine tau isoforms that are likely to reflect different neuronal functions.

## Introduction

In the human central nervous system (CNS), there are six low-molecular-weight tau isoforms ranging from 352 to 441 amino acids in length that are generated by alternative splicing of exons 2, 3 and 10 [1]. This results in isoforms that have 0, 1 or 2 N-terminal inserts (0N, 1N and 2N), and either three (3R) or four (4R) microtubule-binding domains [2]. Tau is developmentally regulated, with the fetal isoform being the shortest [3,4]. In adult brain tissue, the 3R and 4R isoforms exist at an equimolar ratio. This ratio is maintained in the insoluble tau filaments that characterize the neurofibrillary tangles (NFTs) in Alzheimer’s disease (AD) [5]. Different from the human brain, only 4R isoforms have been reported in the adult mouse brain, whereas in the embryo the major isoform is 0N3R. At postnatal day 6 (P6), most tau is still 0N3R, with some 0N4R, but by P90, only 4R tau is present, with 0N4R being the major species [6]. 

 Most studies to date have investigated the differences between the 3R and 4R isoforms rather than the impact of the amino-terminal inserts on tau function. For example, developmental and species-speciﬁc variations in the expression of 3R and 4R tau have been reported within the frontal cortex and hippocampus [6]. In a pathological setting, tau can form aggregates in neurodegenerative diseases such as AD or frontotemporal dementia (FTD), and a distortion of the 3R:4R ratio is known to cause FTD, indicating that 3R and 4R tau must have different functions [7,8]. 4R isoforms interact with microtubules more strongly than 3R isoforms and are more efficient at promoting microtubule assembly [9,10]. Using video microscopy to assess the growing and shortening dynamics of microtubules, it was also found that 4R tau suppresses the shortening rate, whereas 3R tau had little or no detectable effect. Similarly, 3R tau had no effect on the length reduction during a shortening event, whereas 4R tau caused a strong reduction of this parameter [10]. When the interaction of tau and the Src kinase Fyn was investigated *in vitro* using surface plasmon resonance, this revealed a 20-fold stronger interaction of the SH3 domain of Fyn with 3R tau compared with 4R tau [11].  

 What is then known about the role of the amino-terminus and the two alternatively spliced exons that in the adult mouse brain differentiate the three 4R tau isoforms? Early studies showed that the amino-terminal domain (which is also known as the projection domain) is capable of mediating interactions between tau and the plasma membrane in a phosphorylation-dependent manner [12,13]. Tau interacts mainly with its seventh PXXP motif located in the amino-terminal domain with the SH3 domain of Fyn and other Src kinases [14,15]; tau phosphorylation at Tyr18 mediates the interaction with the SH2 domain of Fyn [16]. How these two interactions affect each other in an *in vivo* context is only partly understood. As far as the rate of tau's release into the extracellular space is concerned, this is influenced by the tau isoform [17]. Cells that express tau isoforms without both the amino-terminal exons 2 and 3 (0N3R and 0N4R) had a similar ratio of extracellular to intracellular tau, which was lower than for 2N3R and higher than for 2N4R. Also, as mentioned above, segments encoded by exons 2 (and 10) promote tau aggregation, whereas the segment encoded by exon 3 depresses it [18]. 

 Collectively, these findings strongly indicate that the amino-terminus of tau has an important role in neurodegenerative disorders [19]. These lines of evidence demonstrate a crucial role for the amino-terminus in tau aggregation, spreading, dendritic localization and signaling; however, information on the distribution of the tau isoforms and a detailed insight into their function in a physiological setting is largely lacking. Here we present a detailed immunohistochemical and Western blot analysis of wild-type mice of three age groups, using a set of tau-specific antibodies including newly generated isoform-specific antibodies (01, 1N and 2N) for tau.

## Materials and Methods

### Mice

BALB/c mice were used for antibody preparation. For Western blotting and immuno-histochemistry, C57BL/6J mice were used. The animal experimentation was approved by the Animal Ethics Committees of the University of Sydney (approval number K00/1-2009/3/4914) and the University of Queensland (approval number QBI/027/12/NHMRC).

### Antibody generation

The following peptides were obtained from Scilight Biotechnology LLC (Beijing, China) to generate antibodies specific for total, 0N, 1N and 2N murine tau:

Total tau (M): RVASKDRTGNDEKK (aa 115-128, encoded by exon 5);
0N tau (0N): DMDHGLKAEEAGIG (aa 27-40, bridging exons 1 and 4);
1N tau (1N): DAKSTPTAEAEEAG (aa 54-67, bridging exons 2 and 4); and
2N tau (2N): TEIPEGITAEEAGI (aa 84-97, bridging exons 3 and 4).

For conjugation, a cysteine residue was added to the carboxy-terminus of the peptides followed by linkage to keyhole limpet hemocyanin (Sigma, St Louis, MO, USA) and bovine serum albumin (BSA, Sigma), using 4-maleimidobutyric acid N-hydroxysuccinimide ester (GMBS, Sigma) and a peptide: protein ratio of 50:1. For each antigen, five BALB/c mice were subcutaneously injected with 50 µg of the corresponding peptide mixed with Complete Freund’s adjuvant (Sigma). 3 weeks later, the mice were boosted using Incomplete Freund’s adjuvant (IFA), and after a further 7 days, were bled for the first time to determine the antibody titer. Injecting the peptide/IFA mixture 3 times, at 3-week intervals, boosted the immune response. 

### Titer measurements

To determine titers, blood was taken from the tail vein and serum isolated by centrifugation for enzyme-linked immunosorbant assay (ELISA) [20]. In brief, 96-well Microlon plates (Greiner, Frickenhausen, Germany) were coated overnight with 5 µg/mL of the relevant peptide-BSA complex in ELISA coating buffer (1.50 mM carbonate/bicarbonate buffer, pH 9.6), washed and then blocked with 10% fetal bovine serum (FBS, Invitrogen, Carlsbad, Co, USA) in PBS-T solution (3.2 mM Na_2_HPO_4_, 0.5 mM KH_2_PO_4_, 1.3 mM KCl, 135 mM NaCl, 0.05% Tween 20, pH 7.4) for 2 h at room temperature. Serial dilutions of sera were incubated for 2 h at room temperature and antigen-specific antibodies were detected with horseradish peroxidase (HRP)-coupled anti-mouse antibodies (1:5000; Santa Cruz, Dallas, TX, USA) and SIGMAFAST™ OPD (Sigma). The color reaction was stopped by adding 2 M sulfuric acid, and measured at 490 nm in a Benchmark plus plate reader (BioRad, Hercules, CA, USA). Titers were considered as the highest dilution with a signal to noise ratio > 2.1.

Those sera that revealed high antibody titers by peptide ELISA (>1:72,000) were tested by Western blotting to determine whether they reacted with the appropriate tau isoform(s), using dephosphorylated mouse tau protein. The mice were then sacrificed and the sera collected for subsequent use. More specifically, blood was collected by cardiac puncture and then incubated for 2 h at room temperature followed by an overnight incubation at 4 °C. On the next day the sera were skimmed. The generated polyclonal antibodies were named M(ouse), 0N, 1N and 2N, respectively. 

### Preparation of recombinant tau

pRc/CMV plasmids containing cDNAs for the three murine tau isoforms were a kind gift of Dr. Gloria Lee and are referred to as Mtau10 (0N4R), Mtau210 (1N4R) and Mtau2310 (2N4R), respectively. Forward primer ATGGCTGACCCTCGCCAG and Reverse primer TCACAAACCCTGCTTGGCCAA were used to clone the murine tau cDNAs into the pGEX4t1 plasmid (GE Healthcare, Barrington, IL, USA). Recombinant tau protein was obtained in E. coli (One Shot® BL21-AI™, Invitrogen) using a modified version of a previously published protocol [21]. The bacterial suspension was pelleted at 3,000g for 10 min at 4 °C. The pellet was weighed and resuspended at 10 mL/g pellet in ice-cold PBS (pH7.4) containing 0.1% Complete protease inhibitor (Complete Mini, Roche Applied Science, Basel, Switzerland). The suspension was sonicated for 5 min on ice and Triton X-100 (T8787; Sigma) was added to a final concentration of 1%, followed by incubation at 4 °C on a shaker. After shaking for 30 min, the suspension was centrifuged at 12,000g for 10 min at 4 °C. The supernatant was collected for purification using GSTrap 4B columns following the manufacturer’s guidelines (GE Healthcare). 

### Protein extraction and dephosphorylation

Organs and sub-regions of the brain were dissected and immediately snap-frozen and stored at -80 °C until required. Subcellular protein fractionation was done using the Subcellular Protein Fractionation Kit for Tissue (Thermo Scientific, Melbourne, VIC, Australia, cat# 87790) that allows to obtain cytoplasmic, membrane, soluble nuclear, chromatin-bound and cytoskeletal proteins. To prepare samples for Western blot analysis, samples were homogenized in 10 µl/mg RAB high-salt buffer (0.1 M MES, 1 mM EGTA, 0.5 mM MgSO_4_, 0.75 M NaCl, 0.02M NaF, and 0.1% Complete protease inhibitor (Complete Mini, Roche Applied Science)), using a plastic pistil (Eppendorf, Hamburg, Germany), followed by passing the extract through a 29G insulin needle (Terumo, Piscataway, NJ, USA). The homogenate was then centrifuged at 40,000 g for 40 min at 4 °C. The supernatant was taken, boiled for 5 min, and centrifuged at 13,000 g at 4 °C for 20 min. The pellet was discarded and the supernatant (labeled ‘RAB’) used for subsequent Western blot analysis. Protein dephosphorylation was carried out as described previously [22]. In brief, RAB samples and subcellular fractionation samples were dialyzed in 50 mM Tris–HCl (pH 7.5) overnight and then dephosphorylated with 20 U/μL lambda protein phosphatase (New England Biolabs, Hitchin, UK) for 3 h at 30 °C. Reactions were terminated by adding 4 x sodium dodecyl sulphate–polyacrylamide gel electrophoresis (SDS–PAGE) sample buffer (200 mM Tris-HCl, pH 6.8, 8% SDS, 40% glycerol, 4% β-mercaptoethanol, 50 mM EDTA, 0.08% bromophenol blue), and heating for 5 min at 95 °C, followed by a short centrifugation. The supernatant was then loaded onto an SDS-PAGE gel and used for Western blot analysis. 

### Western blotting

Western blotting was carried out as described previously [23], loading 10 μg of protein extract per lane. However, when assessing the subcellular fractions, there was an enrichment of nuclear proteins in the corresponding fraction because the nuclei had been resuspended in a smaller volume of extraction buffer. There was also an additional enrichment when the samples were heated for dephosphorylation, because nuclear proteins other than tau are less stable at elevated temperature. Proteins were separated by SDS-PAGE (Tetracell, BioRad) and electro-transferred onto nitrocellulose membranes (Hybond, GE Healthcare). The membranes were blocked with 5% BSA in 1× TBST (Tris-Buffered Saline with 1% Tween 20, pH7.4) for one hour at room temperature. Primary antibodies were diluted in 5% BSA/TBST and incubated overnight at 4 °C. In addition to the tau antibodies generated in this study, the following primary antibodies were used: tau5 (Invitrogen, Cat# AHB0042, 1:1000, raised against amino acids 210-241 of human tau), Dako tau (Dako, Glostrup, Denmark, Cat# A002401-2, 1:5000), Tau13 (Abcam, Cambridge, MA, USA, Cat# ab24634, 1:1000), RD3 (Millipore, Billerica, MA, USA, Cat# 05-803, 1:1000), RD4 (Millipore, Cat# 05-804, 1:1000), GAPDH (Chemicon, Millipore, Cat# AB2302, 1:1000), GLT-1 (Alpha Diagnostic, San Antonio, TX, USA, Cat# GLT11-S, 1:1000), hnRNP-A1 (Sigma, Cat# R4528-200U, 1:1000), histone H2A (Millipore, Cat# 07-146, 1:1000), and GFAP (Sigma, Cat# G3893-.2ML, 1:1000). Bands were visualized in a VersaDoc 4000 imaging system (BioRad), using horseradish peroxidase (HRP)-coupled secondary antibodies (Santa Cruz), and the Luminata^TM^ Crescendo Western HRP substrate (Millipore). Band intensities were determined with Quantity One 1-D software v4.6 (BioRad).

### Immunohistochemistry

Immunohistochemistry was carried out as described previously [24]. P0 (postnatal day 0), 2 week- and 2 month-old C57Bl/6 mice were transcardially perfused with 1× PBS, followed by post-fixation in 4% paraformaldehyde overnight at 4 °C. On the following day, paraformaldehyde was exchanged for 70% ethanol. The fixed tissue was dehydrated through an ascending series of ethanol and xylol, embedded in paraffin using an Excelsior tissue processor (Thermo Scientific) and sectioned at 5 µm. The sections were rehydrated step-wise before antigen retrieval was performed in a RHS-1 microwave vacuum histoprocessor (Milestone, Carlsbad, CA, USA) in pre-warmed 10 mM citrate buffer (pH 5.8) for 7 min at 120 °C and then cooled on the bench-top for 15 min. For standardization, all stainings were carried out in Shandon Sequenza racks (Thermo Scientific). After blocking with PBS containing 3% heat inactivated goat serum and 5% BSA for 1 h at room temperature, sections were incubated over night at 4 °C with the primary antibodies in blocking buffer. Primary antibodies were used as follows: Tau5 (pan-Tau, Invitrogen, 1:50), Dako tau (pan-Tau, Dako, 1:200), our newly generated antibodies M (pan-tau), 0N, 1N and 2N (all 1:50). 4, 6-diamidino-2-phenylindole (DAPI, Invitrogen) was used to visualize nuclei. Alexa 555- and 488-coupled secondary antibodies (Invitrogen) were used to detect binding of the primary antibodies. Pre-absorption was done using the SulfoLink immobilization kit for peptides (Thermo): In brief, 0.2mg total tau, 0N, 1N and 2N tau peptide was coupled to 0.2mL SulfoLink beads, respectively. After washing, the coupled SulfoLink beads were incubated with M, 0N, 1N and 2N antibodies, respectively, for 1 h at room temperature. The supernatants were collected for pre-absorption staining after centrifugation at 1,000g for 1 min at room temperature. Pictures were taken with a BX51 fluorescence microscope equipped with a DP70 CCD color camera (Olympus, Center Valley, PA, USA).

## Results

### Generating isoform-specific antibodies for murine tau

**Figure 1:**
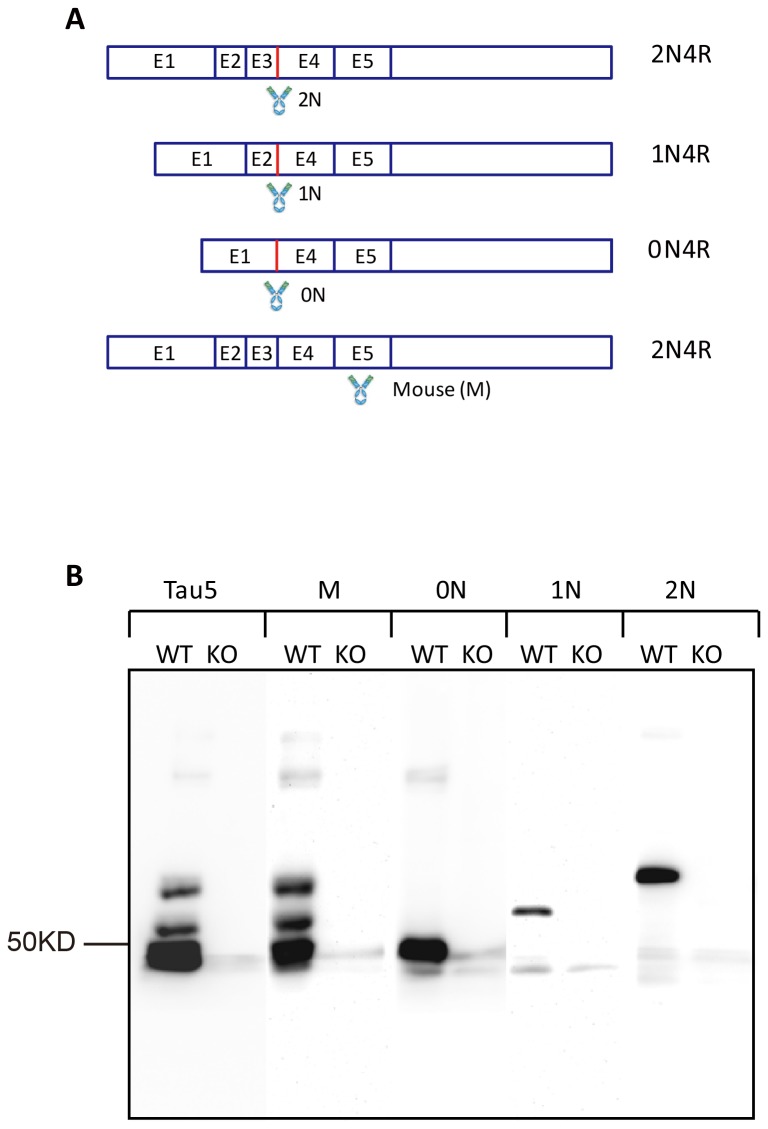
Raising tau isoform-specific antibodies. (**A**) Schematic representation of the exon structure of the MAPT locus that encodes murine tau. Alternative splicing of exons 2, 3, and 10 generates the three isoforms 0N4R, 1N4R, and 2N4R that are present in the adult murine brain. The scheme shows the location of the epitopes that were used to raise specific antibodies for 0N, 1N and 2N murine tau, as well as for total murine tau (M), without cross-reactivity with human tau. (**B**) Western blot analysis of RAB-soluble tau extracts obtained from brains of 2-months old wild-type (WT) mice, with stripes probed separately with Tau5, M (total mouse tau) and the murine tau isoform-specific antibodies 01, 2N, and 2N reveals their specificity. Tau knock-out (KO) tissue was included as negative control.

Alternative splicing generates the three major tau isoforms in the adult mouse brain (Figure 1A). These isoforms contain either no (0N), one (1N) or two (2N) N-terminal inserts. Splicing of exons 1 and 4 generates the 0N isoform, that lacks exons 2 and 3 and hence the first and second N-terminal inserts. To generate a 0N-specific antibody, we used a peptide encompassing the flanking amino acid sequences encoded by exons 1 and 4 (Figure 1A). Applying the same principle, we raised antibodies for the 1N isoform (for which the amino acids encoded by exons 2 and 4 are uniquely joined), and 2N (for which the amino acids encoded by exons 3 and 4 are uniquely joined). In addition, we generated a pan-tau antibody (labeled M) for reactivity with all forms of murine tau. We first tested the antibodies by Western blotting using recombinant tau (data not shown) followed by Western blotting using RAB brain fractions obtained from wild-type, tau knock-out [25], and human P301L mutant tau expressing pR5 mice [26]. Compared with the pan-tau antibody Tau5 that is reactive with both human and murine tau isoforms, and Tau13 that has a higher affinity for human tau, the murine tau-specific M antibody reacted with all three mouse isoforms, but not human tau, and failed to detect bands in extracts obtained from tau knock-out brains (Figure 1B). To determine the specificity of the four newly generated antibodies, we also performed Western blotting of RAB-soluble brain extracts from wild-type and tau knock-out mice. This revealed a smear as expected for tau, which is known to be phosphorylated at multiple sites even under physiological conditions (data not shown). Upon dephosphorylation, Tau5 revealed three bands representing the three major adult CNS tau isoforms, 0N, 1N and 2N, in wild-type and not, tau knock-out mice (Figure 1B). Our M antibody revealed the same pattern as Tau5, whereas the three isoform-specific antibodies reacted only with the respective isoforms as anticipated (Figure 1B).

### Tau is expressed at high levels in brain

**Figure 2:**
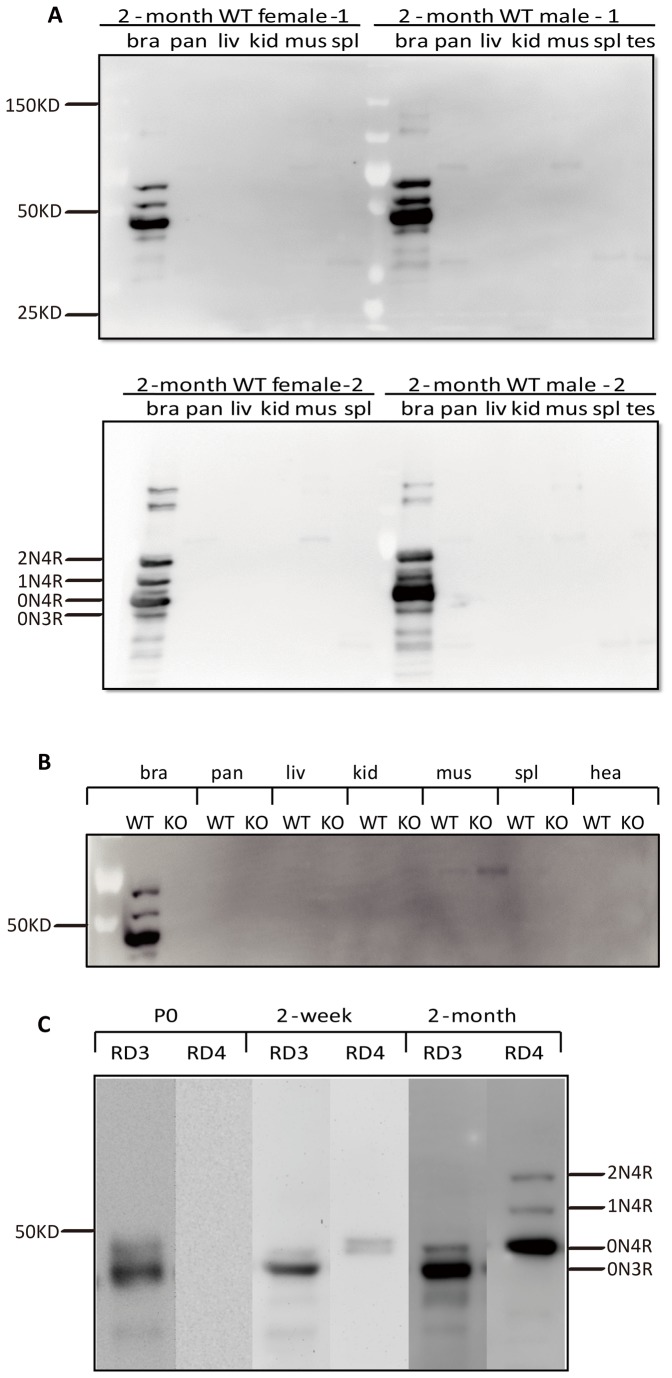
Western blot analysis of dephosphorylated extracts obtained from dissected tissues of 2-month old mice using the Tau5 antibody. (**A**) Analysis of wild-type (WT) mice; brain (bra), pancreas (pan), liver (liv), kidney (kid), muscle (mus), spleen (spl), testis (tes), and heart (hea). (**B**) Inclusion of tau knock-out (KO) tissue. Molecular weight and isoforms are indicated. (**C**) Western blot analysis of dephosphorylated brain extracts from 2-month old, 2-week old and P0 WT mice using the 3R- and 4R-specific antibodies RD3 and RD4, respectively. Note: The relatively intensities of RD3 and RD4 cannot be used to deduce the relative levels of the 3R and 4R isoforms. Instead, the Tau5 pattern is informative.

Previous reports suggested that in rodents tau is not only expressed in brain but also in several peripheral tissues at fairly high levels [27-29]. To obtain a comprehensive picture of tau isoform distribution under physiological conditions, we used both commercially available, pan-specific as well as our novel isoform-specific antibodies for Western blotting and immunohistochemistry. Throughout the study we always used two male and two female C57Bl/6 wild-type mice. We first set out to determine in which tissues the three murine tau isoforms are expressed, by generating RAB extracts from total brain, pancreas, liver, kidney, muscle, spleen and testis, followed by dephosphorylation. We found by Western blotting using Tau5, and within the sensitivity of the assay, that prominent expression of tau was restricted to the brain in 2 month-old mice (Figure 2A). The major isoform was 0N4R, with the 1N4R and 2N4R bands showing much lower signal intensities. Trace amounts of 0N3R were also detected. Tau bands within the size range of 0N3R to 2N4R were not found in any of the other tissues examined. A faint band at around 100 kDa was inconsistently found in muscle and pancreas and therefore may be considered to be background staining. As a control, we included tau knock-out mouse brain extracts that also included heart (Figure 2B). A knock-out control had not been included in previous studies that had suggested expression of tau in major tissues other than the brain, such as kidney, testis or muscle, while on the other hand the authors of this study indicated substantial cross-reactivity of their antibodies with MAP2C and other proteins [27]. 

 We also determined the levels of the 3R and 4R tau isoforms using the antibodies RD3 and RD4, respectively (Figure 2C). Unlike at 2 weeks and 2 months of age, 3R tau was the only isoform expressed at P0, with no 4R tau detectable. Although we did detect tau at 2 months using the RD3 antibody, levels compared to 4R tau were low as is evident from Figures 2A and B. Is has to be noted that relative ratios of 3R and 4R tau can not be determined by comparing band intensities of the RD3 and RD4 blots because the affinities of these antibodies and the corresponding epitopes differ. Instead, staining with Tau5 that detects both 3R and 4R tau reveals that levels of 3R tau in adult tissue are indeed very low.

### The 1N isoforms is over-represented in the pituitary gland and under-represented in the olfactory bulb

**Figure 3:**
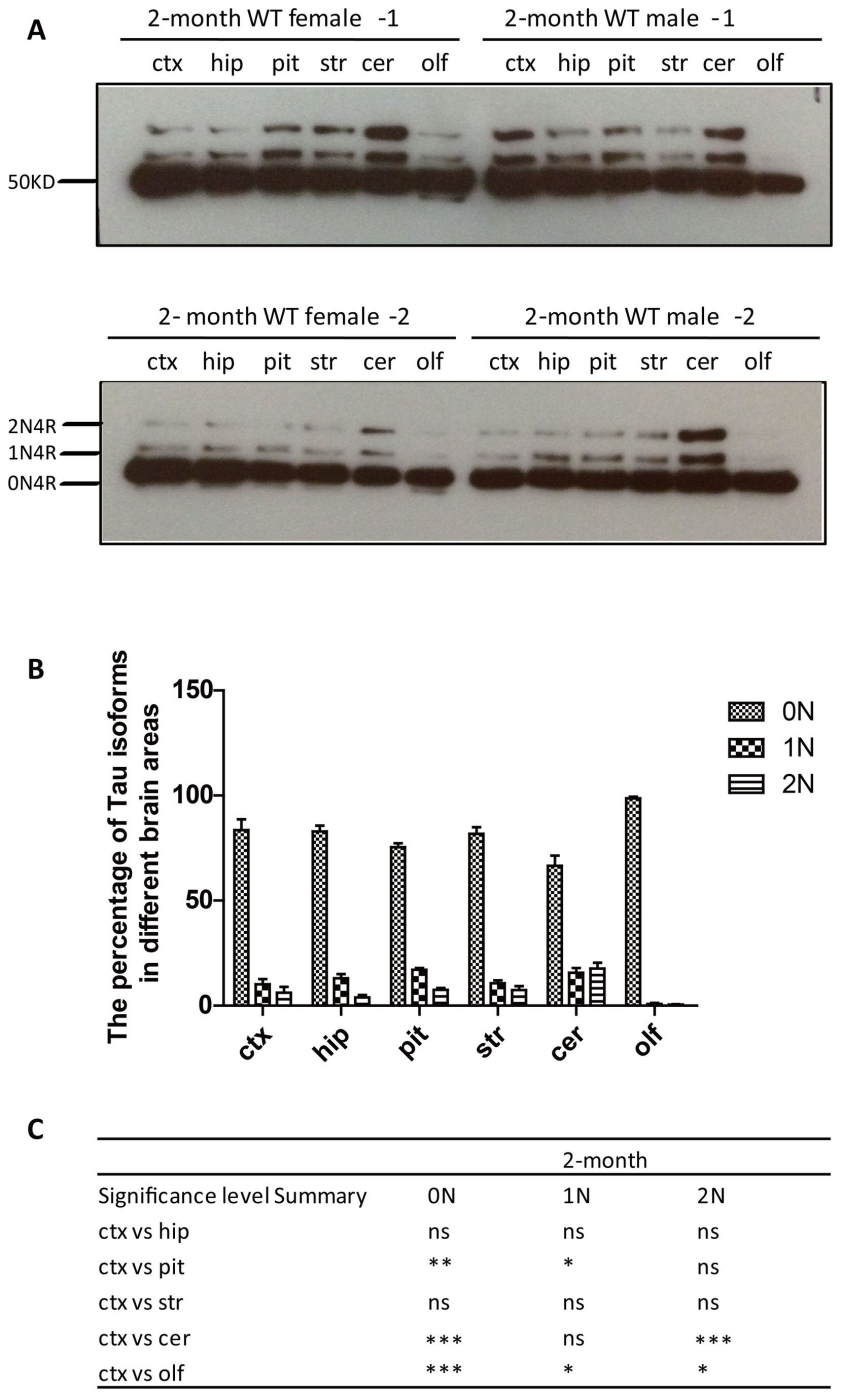
Western blot analysis of dephosphorylated samples derived from different brain areas of 2-month old WT mice using the Tau5 antibody. (**A**) Brain tissues: cortex (ctx), hippocampus (hip), pituitary gland (pit), striatum (str), cerebellum (cer) and olfactory bulb (olf). (**B**) Relative levels of tau isoforms in the different brain areas. (**C**) Significance analysis using two-way ANOVA. The significance level is calculated by comparing to cortex. *, P< 0.05, **, P< 0.01, ***, P< 0.001, and ns, not significant. Error bars represent the standard error of the mean (SEM).

To determine whether 0N, 1N and 2N are differentially expressed in the brain, we analyzed the cortex, hippocampus, pituitary gland, striatum, cerebellum and olfactory bulb dissected from two male and two female 2 month-old wild-type mice and found that the three isoforms were expressed in all brain areas examined (Figure 3A). Determining differences in total tau levels across brain areas is complicated by the fact that dephosphorylation requires pre-heating, followed by centrifugation, with tau going into the supernatant and markers such as GAPDH or actin into the pellet. Our intention, however, was not to determine the comparative levels of total tau in different brain areas but rather to determine the relative distribution of the three isoforms within a particular brain area. We found the following values for 0N: 83.6% of total tau in the cortex, 82.9% in the hippocampus, 75.4% in the pituitary gland, 81.8% in the striatum, 66.6% in the cerebellum and 98.7% in the olfactory bulb. 1N comprised 10.2% of total tau in the cortex, 13.1% in the hippocampus, 17.0% in the pituitary gland, 10.7% in the striatum, 15.7% in the cerebellum and 0.8% in the olfactory bulb, and 2N comprised 6.2% of total tau in the cortex, 3.9% in the hippocampus, 7.5% in the pituitary gland, 7.5% in the striatum, 17.7% in the cerebellum and 0.5% in the olfactory bulb (Figure 3B). The statistical analysis revealed, that the 1N isoform is significantly over-represented in the pituitary gland, that the 2N isoform is enriched in the cerebellum, and that the 1N and 2N isoforms are underrepresented in the olfactory bulb (Figure 3C). 

### The 1N isoform is over-represented in the soluble nuclear fraction

**Figure 4:**
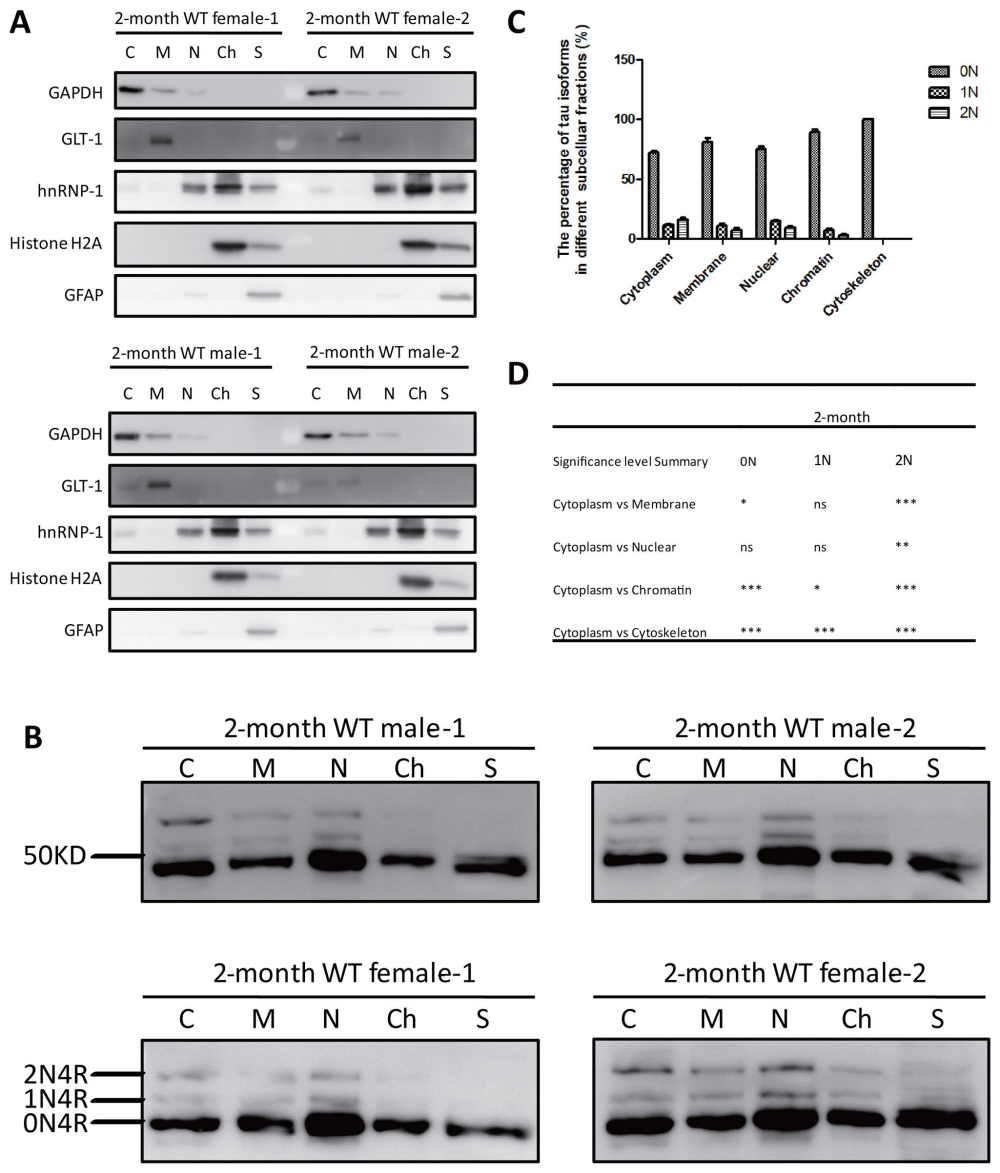
Subcellular fractionation of brains from 2-month old WT mice. (**A**) Relative purity of the cytoplasmic (C), membrane (M), nuclear (N), chromatin-bound (nuclear) (Ch) and cytoskeletal (S) fractions were confirmed using the following antibodies: GAPDH (for C), GLT-1 (for M), hnRNP-1 (for N), histone H2A (for Ch), and GFAP (for S). (**B**) Western blot analysis of dephosphorylated brain fractions obtained from 2-month old WT mice using the tau-specific Tau5 antibody. (**C**) Relative ratio of the three tau isoforms in the five fractions. (**D**) Significance analysis using two-way ANOVA. The significance level is calculated by comparing to the cytoplasmic fraction. *, P< 0.05, **, P< 0.01, ***, P< 0.001, and ns, not significant. Error bars represent the standard error of the mean (SEM).

To determine whether subcellular fractionation would reveal differences in distribution between the three isoforms, we analyzed mouse brains at 2 months, 2 weeks and at postnatal day 0 (P0). We obtained a total of five fractions: cytoplasmic, membrane, soluble nuclear, chromatin-bound, and cytoskeletal. We first determined, using 2 month-old mice, that the fractionation protocol yielded the expected fractions, using the following markers: GAPDH (as a marker for the cytoskeletal fraction), GLT-1 (for membrane), hnRNP-1 (for soluble nuclear), histone H2A (for chromatin-bound), and GFAP (for cytoskeletal), using two male and two female brains each (Figure 4A). Having confirmed that our extraction yielded reasonably pure subcellular factions, we next determined the distribution of the tau isoforms in these fractions (Figure 4B), followed by quantification (Figure 4C) and a statistical analysis (Figure 4D). This revealed that for the cytoplasmic fraction, the 0N:1N:2N ratio was 72:12:16, for membrane it was 81:12:7, for soluble nuclear 75:15:10, for chromatin-associated 90:7:3, and for cytoskeletal 100:0:0. Together, this shows that the 1N isoform is under-represented in the chromatin-associated and cytoskeletal fractions and over-represented in the soluble nuclear fraction. The 2N isoform, in comparison, is under-represented in the membrane, soluble nuclear, chromatin-binding and cytoskeletal fractions (Figure 4D). How can the virtual absence of 1N and 2N in the cytoskeletal fraction be explained? While in principle, tau may dissociate from microtubules during the subcellular fractionation process, this was not generally the case as 0N could be easily obtained whereas 1N and 2N could not. Conclusions about relative tau levels in the five subcellular fractions cannot be drawn, because in the process of subcellular fractionation and when heating as is the case for dephosphorylation, nuclear proteins, for example, become enriched because nuclei firstly are resuspended in a smaller volume of extraction buffer and secondly, because (tau being an exception) they are less stable with heating. What can, however, be compared are the ratios of the tau isoforms within any particular fraction.

**Figure 5:**
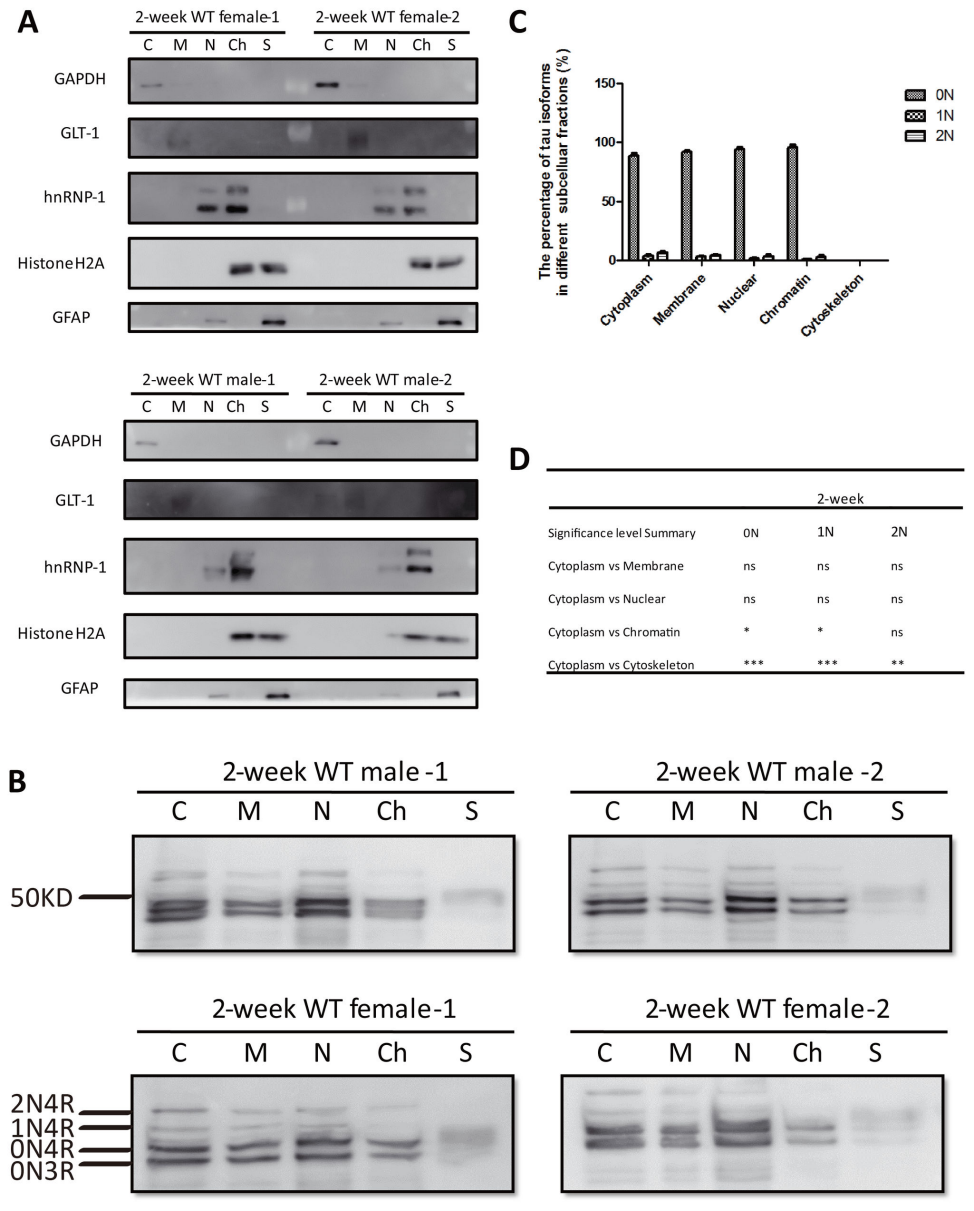
Subcellular fractionation of brains from 2-week old WT mice. (**A**) Western blot analysis using the same subcellular markers as used in Figure 4. (**B**) Western blot analysis of dephosphorylated brain fractions obtained from 2-week old WT mice using Tau5. Note: At 2 weeks, there is also expression of the fetal (3R) isoform. The 0N3R and 0N4R isoforms have been assessed 'collectively'. (**C**) Relative ratio of the three tau isoforms in the five fractions. (**D**) Significance analysis using two-way ANOVA.

 We next analyzed 2-week-old mice which, in agreement with previous studies [6], revealed the presence of the fetal 0N3R isoform in addition to the three 4R isoforms (Figure 5A,B). By assessing the 0N3R and 0N4R isoforms collectively, we obtained the following 0N:1N:2N ratios: cytoplasmic fraction: 89:4:7, membrane fraction: 92:3:5, soluble nuclear fraction: 94:2:4, and chromatin-binding fraction: 96:1:3 (Figure 5C). Interestingly, the cytoskeletal fraction revealed a smear instead of discrete tau bands, indicating low to no tau in this fraction. This finding may reflect the increased plasticity of the nervous system at this developmental stage, as also evidenced by the high relative levels of 3R tau (Figure 5B). The statistical analysis revealed that the 1N isoform was less represented in the chromatin-associated and cytoskeletal fractions (Figure 5D). 

**Figure 6:**
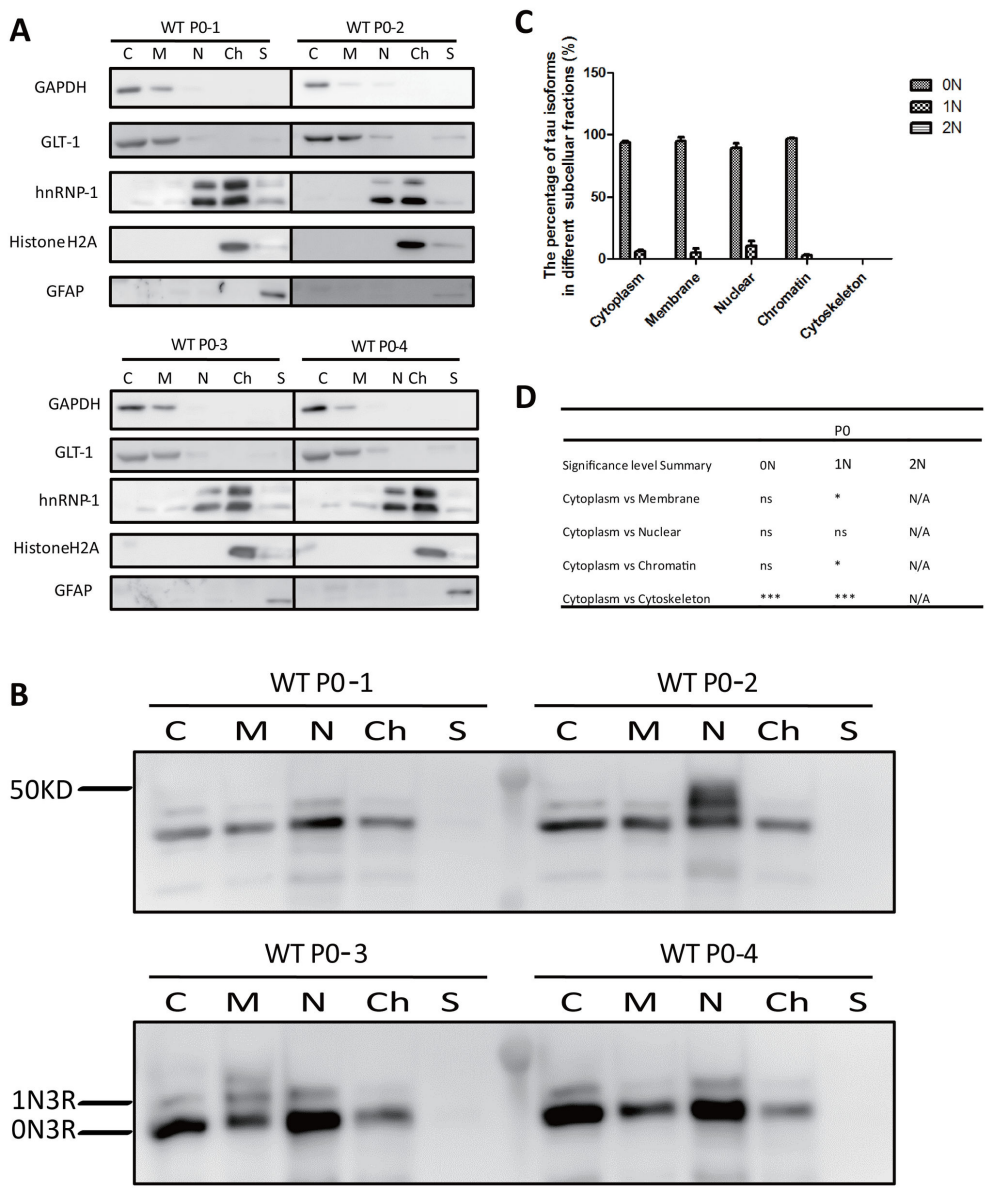
Subcellular fractionation of brains from WT mice at P0. At this stage, 4R isoforms are not expressed. 0N3R is the predominant species, and 2N3R is not detected. (**A**) Western blot analysis using the same subcellular markers as in Figure 4. (**B**) Western blot analysis of dephosphorylated brain fractions obtained from P0 WT mice using Tau5. (**C**) Relative ratio of the three tau isoforms in the fractions. (**D**) Significance analysis using two-way ANOVA.

**Figure 7:**
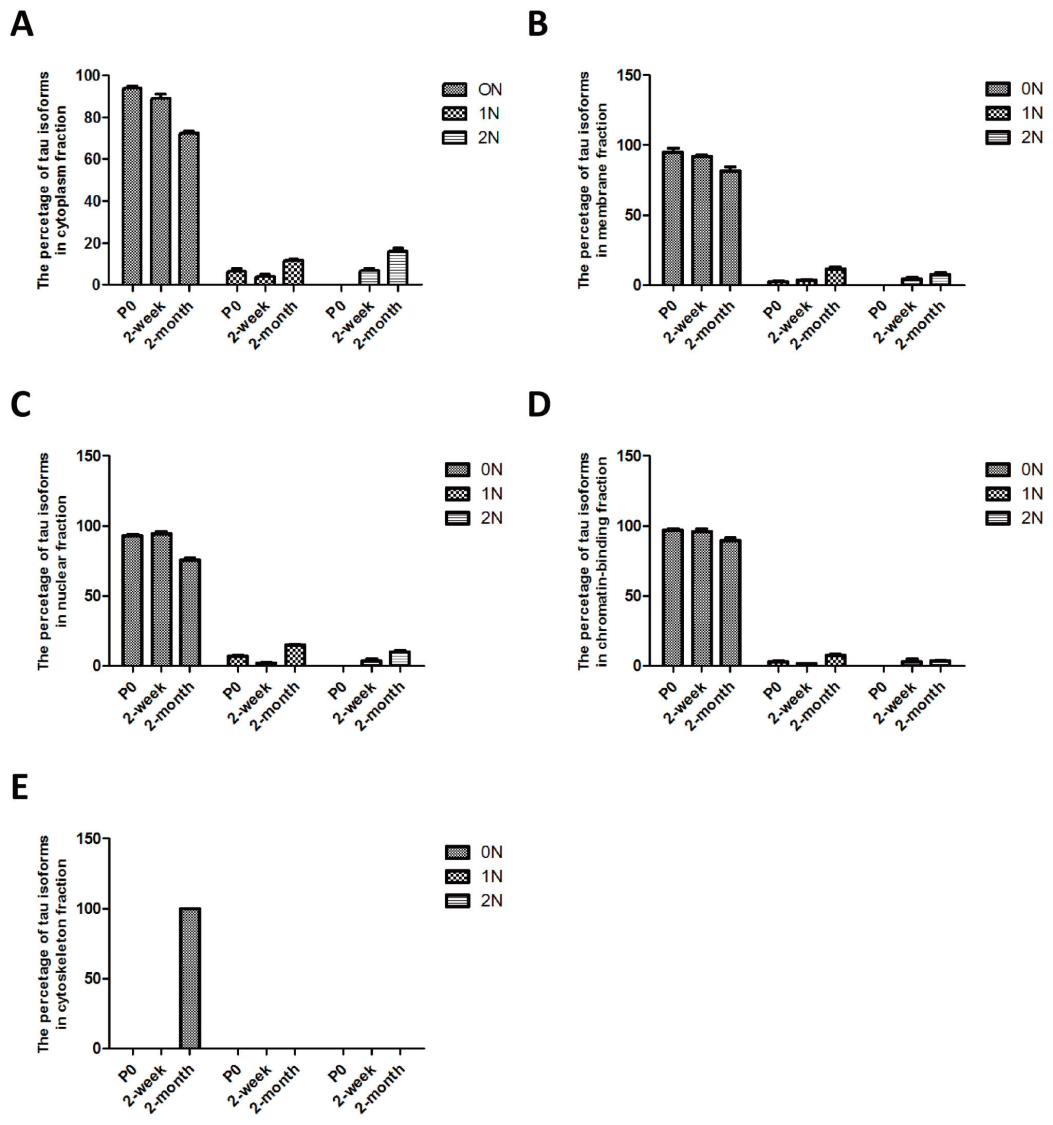
Relative ratio of tau isoforms in five subcellular fractions at P0, 2 weeks and 2 months of age. (**A**) Cytoplasmic, (**B**) membrane, (**C**) soluble nuclear, (**D**) chromatin-bound, and (**E**) cytoskeletal fraction. Error bars represent the standard error of the mean (SEM).

 Finally, we also analyzed P0 brains by subcellular fractionation (Figure 6A). At this stage, 4R isoforms are not expressed, and 0N3R is the predominant species (Figure 2C). We know that the 0N3R isoform detected in Figure 6B is not 1N4R because at P0, the RD4 antibody does not detect any tau. Quantification revealed the following 0N3R:1N3R ratios: cytoplasmic fraction: 94:6, membrane: 95:5, soluble nuclear: 89:11, chromatin-associated: 97:3, but again, not traces of tau were detected in the cytoskeletal fraction (Figure 6B,C). At P0, no 2N3R was detected and again, the 1N isoform was found enriched in the soluble nuclear fraction (Figure 6C). A comparative overview of the findings for the three age groups is shown in Figure 7.

### Immunohistochemistry reveals major differences in localization of the three tau isoforms in mouse brain

**Figure 8:**
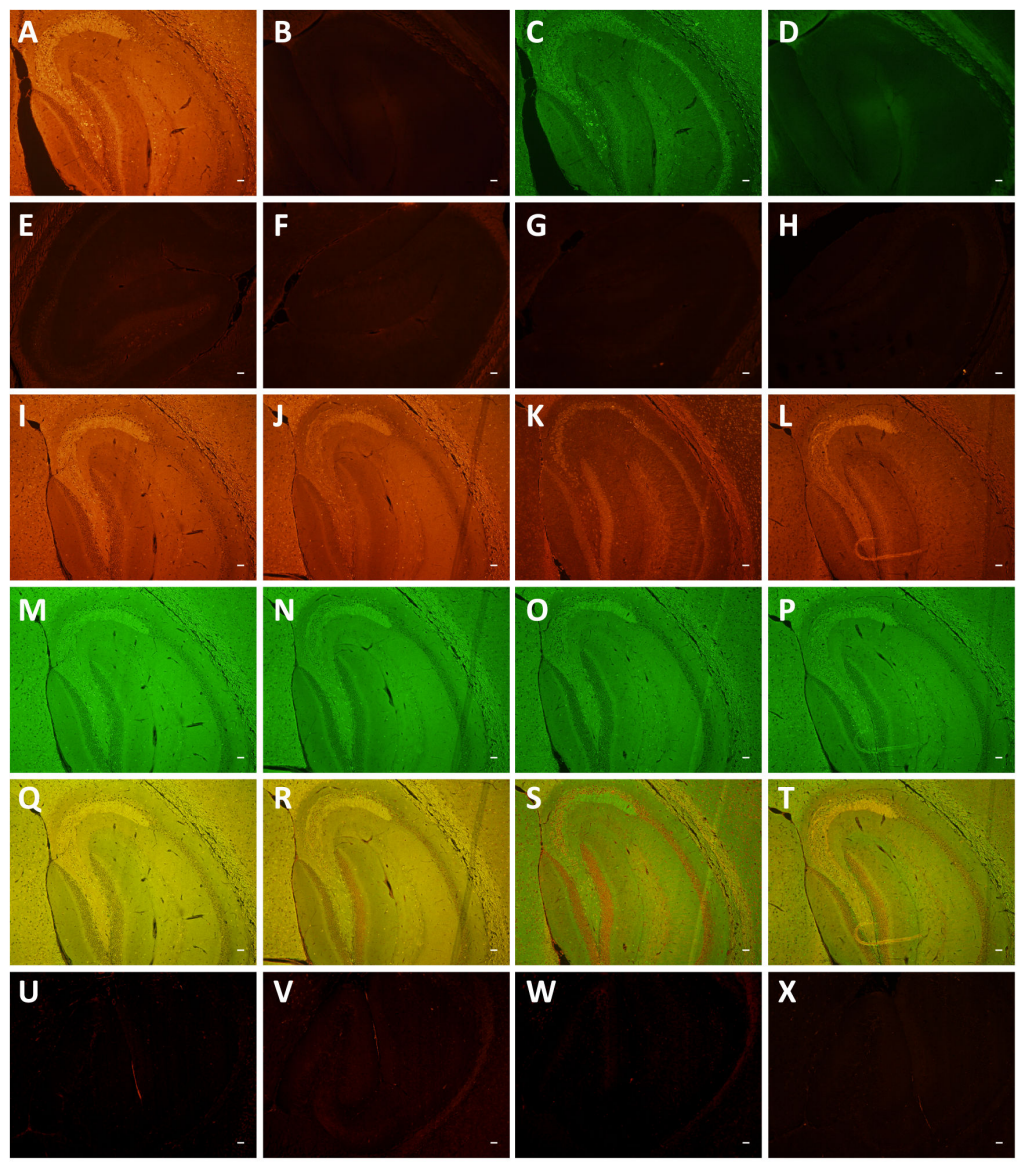
Immunohistochemical analysis of tau isoforms in 2 month-old wild-type mice. (**A**-**D**) Testing of antibodies reveals no bleach-through. Staining of the hippocampus with (**A**) Tau5 and (**C**) Dako tau. Omitting the primary antibodies and reacting the sections only with the secondary antibodies (**B**) Alexa Fluor 555 goat anti-mouse IgG, or (**D**) Alexa Fluor 488 goat anti-rabbit IgG. (**E**-**H**) Pre-absorption with the peptides, with which the corresponding antibodies pan-tau M (**E**), 0N (**F**), 1N (**G**) and 2N (**H**) were generated. (**I**-**L**) Staining with the new antibodies M (**I**), 0N (**J**), 1N (**K**) and 2N (**L**) in red, (**M**-**P**) counter-staining with Dako tau in green, (**Q**-**T**) Merged images. (**U**-**X**) Sections from tau knock-out mice used as negative control for antibodies pan-tau M (**U**), 0N (**V**), 1N (**W**) and 2N (**X**). Scale bar: 50 μm.

**Figure 9:**
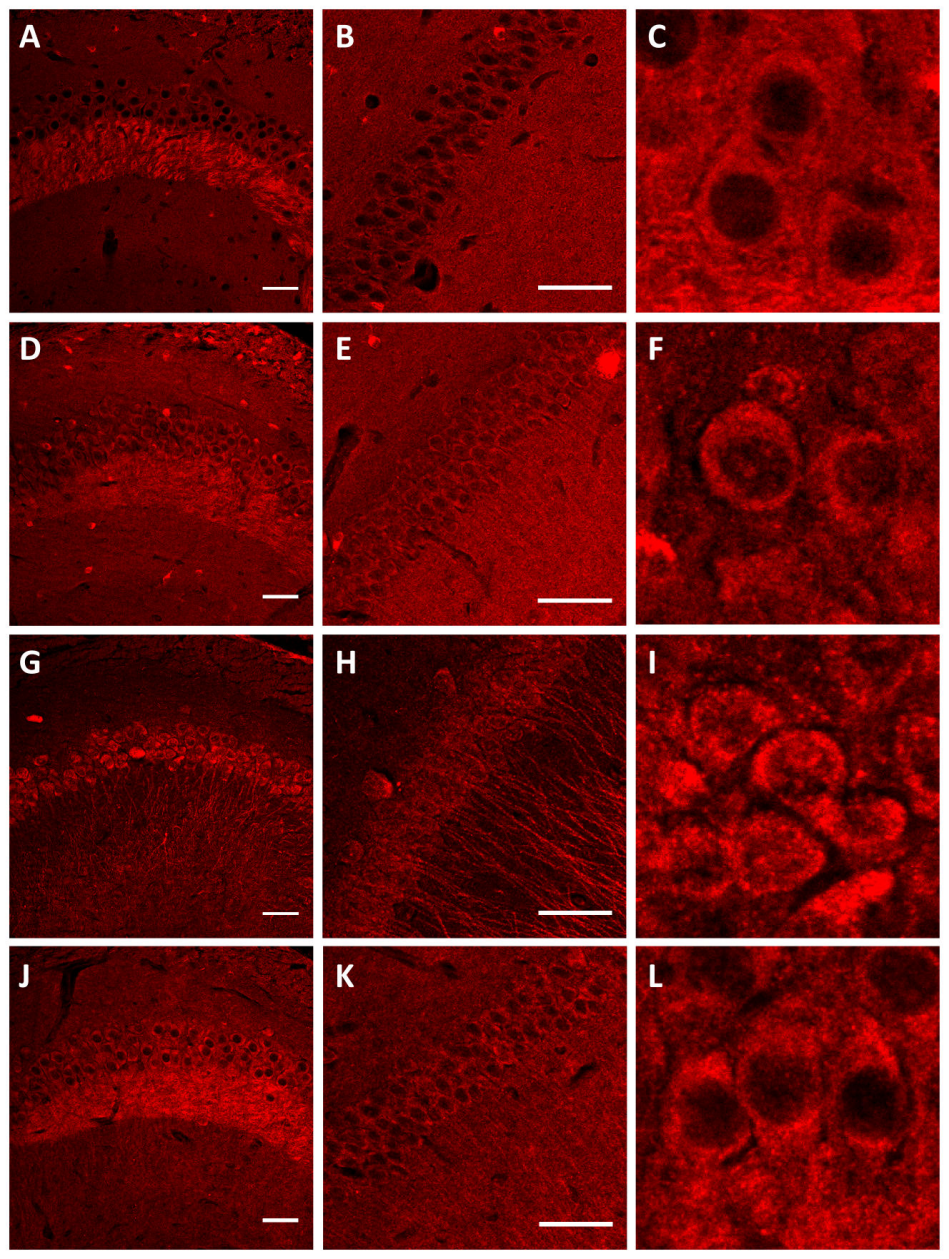
High magnification images of the immunohistochemical analysis of tau isoforms in 2 month-old wild-type mice reveals differences in subcellular localization. Staining with pan-tau M antibody (**A**-**C**), 0N (**D**-**F**), 1N (**G**-**I**) and 2N (**J**-**L**). Close-up of the CA3 region (**A**,**D**,**G**,**J**), CA2 region (**B**,**E**,**H**,**K**) and nuclei in the CA3 region (**C**,**F**,**I**,**L**). Scale bar: 50 μm.

We next determined whether there are differences in the subcellular localization of 0N, 1N and 2N tau in mouse brain using immunohistochemistry. To establish the system, we first tested the total tau-specific antibodies 'Dako tau' and 'Tau5' on brains sections of 2 month-old mice. To assist in the interpretation of our data, we grouped the images according to magnification, low in Figure 8, and high in Figure 9. This revealed an overlapping pattern of 'Dako tau' and 'Tau5', with no bleach-through when the primary antibodies were omitted (Figure 8A-D). We confirmed specificity of the M (murine tau), 0N, 1N and 2N antibodies, and the absence of any background by pre-absorption with the corresponding peptides (Figure 8E-H). We also included sections from tau knock-out mice as negative controls for the reactivity of the four antibodies (Figure 8U-X). Next, we tested the M antibody and found, as expected, that the pattern of M and Dako staining overlapped (Figure 8I,M,Q). The M antibody revealed staining mainly of cell bodies and axons, as shown for the mossy fiber projection in the CA3 region of the hippocampus (Figure 9A). Next, we examined the localization of the 0N, 1N and 2N tau isoforms, again using the hippocampus as a representative brain area. 0N was mainly found in cell bodies and axons as shown for the mossy fiber projection in the CA3 region (Fig. 8JN,R, 9D-F). Nuclei and dendrites were only slightly stained by the 0N antibody, and the staining pattern of 0N largely overlapped with that of Dako tau. The 0N isoform was also found in a few small, unidentified cells (Figure 9D), in agreement with previous findings [28]. Interestingly, 1N was predominately found in the nucleus, in dendrites throughout the hippocampus (shown for CA2), and in the soma (Figures 8K,O,S, 9G-I). The merged image of 1N and Dako tau revealed the lack of axonal expression of 1N in the CA3 mossy fiber region. whereas the 2N isoform was highly expressed in axons as shown for the CA3 mossy fibers, and in cell bodies, with a lower level of expression in dendrites and very slight expression in nuclei (Figure 8L,P,T, J-L). In conclusion, the 0N, 1N and 2N isoforms of tau reveal a distinct subcellular localization as summarized in (Table 1). 

**Table 1:** Subcellular expression patterns of tau isoforms at 2 months of age.

	Staining intensity			
	Soma	Axon	Dendrite	Nucleus
0N	***	***	*	*
1N	***	ND	***	***
2N	***	***	**	*

(***) strong staining, (**) moderate staining, (*) light staining, (without date) not detectable.

**Figure 10:**
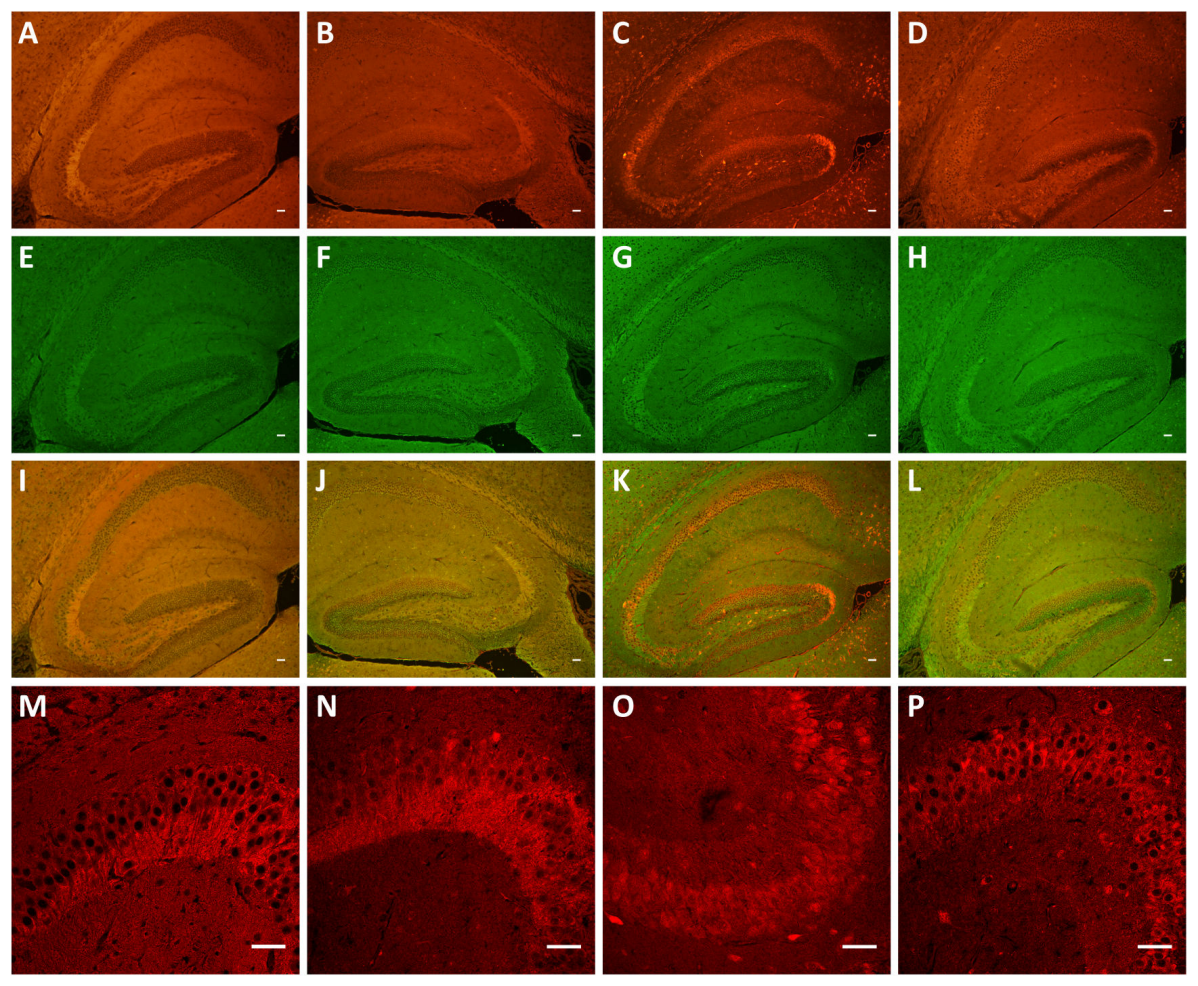
Expression of murine tau in the hippocampus of 2-weeks old WT mice. (**A**-**D**) Staining of the hippocampus with the new antibodies pan-tau M (**A**), 0N (**B**), 1N (**C**), and 2N (**D**) in red, counter-stained with Dako tau in green (**E**-**H**), merged images (**I**-**L**). (**M**-**P**) High magnification images of the hippocampal CA3 region using pan-tau M (**M**), 0N (**N**), 1N (**O**), and 2N (**P**). Scale bar: 50 μm.

**Figure 11:**
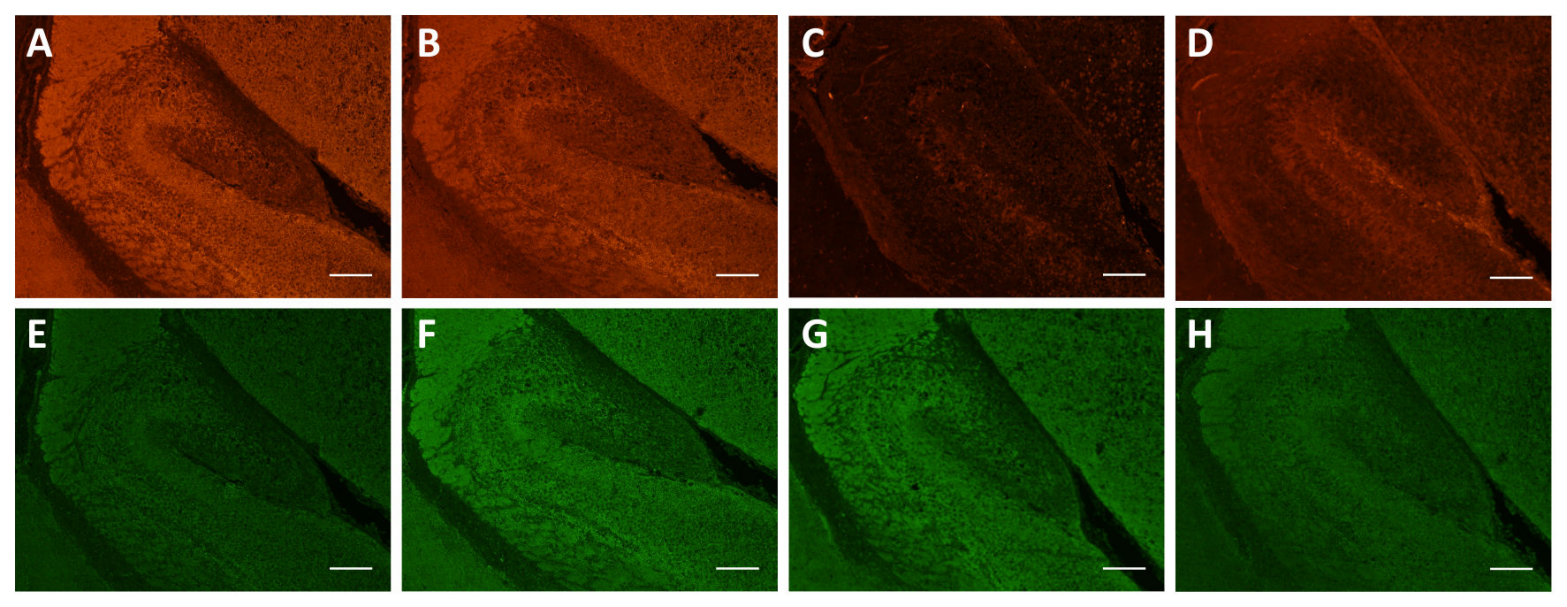
Expression of murine tau isoforms in the hippocampus of WT mice at day P0. At this stage, 0N (0N3R) is the major isoform. (**A**-**D**) Staining with the pan-tau M antibody (**A**), 0N (**B**), 1N (**C**), and 2N (**D**). (**E**-**H**) Counter-staining with Dako tau. Scale bar: 50 μm.

 We next examined the subcellular localization of 0N, 1N and 2N in both 2-week old and newborn (P0) mice. At 2 weeks, tau expression was consistently lower than at 2 months, especially for the 2N isoform (Figure 10, Table 2), in agreement with the Western blot analysis (Figure 5). At P0, the hippocampus is not yet fully developed and tau expression was found to be consistently low (Figure 11). At this stage, 0N (0N3R) was the major isoform, with pronounced staining of cell bodies (Figure 11A). Slight nuclear staining of 1N (1N3R) was also evident (Figure 11C), whereas 2N3R was almost undetectable (Figure 11D). Taken together, the expression of all three tau isoforms showed significant increases from P0 to 2 months of age. 0N was the predominant isoform of tau, and was mainly localized to cell bodies and axons. 1N was highly expressed in the nucleus, cell bodies, and dendrites. 2N, in comparison, was not detected at P0, and in adulthood was mainly found localized to cell bodies and axons. Our data show for the first time the distinct subcellular localization and distribution of the three murine tau isoforms from newborn to adult tissue.

**Table 2:** Subcellular expression patterns of tau isoforms at 2 weeks of age.

	Staining intensity			
	Soma	Axon	Dendrite	Nucleus
0N	***	***	*	*
1N	**	ND	*	**
2N	***	*	*	*

(***) strong staining, (**) moderate staining, (*) light staining, (without date) not detectable.

## Discussion

To provide the foundation for a better understanding for why three murine tau isoforms are expressed in the adult brain, we generated isoform-specific antibodies utilizing the unique joining of amino acids in the amino-terminus of tau for their generation. We also employed commercially available anti-tau antibodies. We addressed the distribution of the three tau isoforms 0N4R, 1N4R and 2N4R in various tissues and brain areas, as well as subcellularly. Whereas on a Western blot a pan-tau-specific antibody does discriminate the three tau isoforms based on their molecular size, isoform-specific antibodies are essential for an immunohistochemical analysis because in this situation, the three isoforms cannot be discriminated with pan-tau-specific antibodies. Using our newly generated isoform-specific antibodies, we found remarkable differences between the three murine tau isoforms. 

 Under normal conditions it is difficult to detect endogenous tau on brain sections (see e.g. for brain in Figure 3B and spinal cord in 4B [30]) [31,32]. It is even more difficult to detect tau in the nucleus, despite the fact that tau has been localized to this subcellular compartment, executing functions that range from protecting DNA to organizing the structure of nucleoli [33,34]. We were therefore surprised to see how strong the nuclear staining was when we employed the 1N tau-specific antibody. Our finding that 1N tau localizes strongly to the nucleus is supported by our subcellular fractionation data, which demonstrated that 1N tau was enriched in the soluble nuclear fraction. The fact that the 1N tau-specific antibody revealed strong nuclear staining but that 1N only constituted a fraction of nuclear tau as determined by Western blotting suggests that, in the nucleus, this isoform is accessible and in a conformation that allows binding of the 1N antibody. In support of this, tau binds to and protects DNA under conditions of heat shock, which would imply that under normal conditions, nuclear tau in the nucleus exists in an unbound form [35]. Together these findings indicate that 1N tau may perform a unique role in the nucleus. While 1N predominantly localizes to the nucleus, our immunochemical data revealed nuclear staining for all three isoforms, which is in agreement with our Western blot analysis. Our fractionation data indicate within the limitations of the fractionation protocol that all three isoforms of tau are in the soluble nuclear fraction.

 What is known about tau's functions in the nucleus? Although tau was primarily described as a microtubule-associated protein with a preferential axonal localization, it has also been observed in the nuclei of both neuronal and non-neuronal cells [36,37]. Nuclear tau binds specifically to AT-rich α-satellite DNA sequences and co-localizes at the border of the nucleolus with nucleolin, a major nucleolar organizer. Moreover, it has been suggested that nuclear tau has an effect on the conformation of the nucleolus [34]. Other studies suggest a protective role for nuclear tau, such as by binding to the minor groove of DNA [38]. Recruitment of tau to the nucleus is determined by tau-DNA binding as unbound tau does not stay in the nucleus, as shown with the DNA-binding antibiotic netropsin [35]. In pathological conditions such as AD that are characterized by the accumulation of insoluble aggregates of tau, the question that arises is how this affects nuclear integrity. Expressing the longest human tau isoform, 2N4R, in SH-SY5Y neuroblastoma cells, causes significant deformity of the nuclear compartment, with extensive lobulations along the nuclear envelope, and alterations of the assembly of the tubulin cytoskeleton, which is modified from a radial organization to a more peripheral and perinuclear distribution in the form of thick rings [39]. It is reasonable to assume that these nuclear changes impair the nuclear pores, leading to altered nuclear transport [40]. This could be one of the reasons why there is a growing list of transcription factors with an abnormal nucleo-cytoplasmic relocalization under neurodegenerative conditions. Specifically, SFPQ, a nuclear factor with a role in splicing and the regulation of gene expression, is depleted from the nucleus and forms cytoplasmic aggregates in tauopathies such as AD and Pick's disease [41]. 

 The 1N isoform, in addition to its nuclear prominence, was also highly expressed in dendrites, similar to what has been reported for Purkinje cells in rats [29]. Unlike 1N tau, 2N mouse tau shows robust cytoplasmic and axonal expression, moderate dendritic expression and a lower level of nuclear expression. Because we found that 2N tau is depleted from the cytoskeletal fraction, this might indicate that it is more likely to be involved in cytosolic functions. As shown in our study and by others, the 0N, 1N and 2N tau isoforms are differentially expressed in the developing and adult brain, revealing subtle differences for the three isoforms. The data complement those of a previous study which found that, at P6, there is mainly 0N3R and only some 0N4R expressed [6]. At around 4 weeks of age, 0N3R is replaced by the 4R isoforms. Between P0 and 2 months of age, we found a reduction of 0N from 94 to 72% and an increase of 1N and 2N from 6 to 12% and 0 to 16%, respectively, which is largely consistent with previous findings [6]. In a pathological context, a fibrillization assay has shown that the presence of sequences encoded by exon 2 (which is present on 1N and 2N tau) promotes tau aggregation, whereas the segments encoded by exon 3 (only found on 2N tau) inhibit tau aggregations [18]. The functional implications remain to be determined but the data suggest that, as for 3R and 4R tau in the human brain, a balance of 1N and 2N needs to be maintained. 

 Tau is mainly an axonal protein, although we have recently found that even under physiological conditions, trace amounts are localized to the dendrite [15]. Our immunohistochemical data show there is pronounced dendritic expression of the 1N and 2N isoforms whereas 0N, the major isoform as shown by Western blotting, is only weakly expressed in the dendrite. One can speculate that the 1N and 2N isoforms preferentially localize to dendrites because they specifically or better interact with dendritic and postsynaptic proteins. One of the synaptically localized proteins is the Src kinase Fyn that is targeted in a tau-dependent manner to the dendritic compartment utilizing a PXXP motif localized to the amino-terminus of tau [11,15]. We observed robust axonal staining as shown for the 0N and 2N isoforms in the CA3 mossy fiber region, whereas there was no detectable axonal staining revealed for the 1N isoform. One possible explanation is that 1N may not be significantly involved in microtubule stabilization. A role for 0N3R in adult neurogenesis has been suggested in rats [28]. In this study, doublecortin-positive neurons were separately shown to be 0N- and 3R-positive. Because both the 0N and 3R antibodies are monoclonal, the essential control of double staining could not be done in order to substantiate the claim that these cells are indeed strongly expressing 0N3R tau. Pre-absorption with the corresponding peptide was also not done. A third control (that of a tau knock-out) could not be used because (different from our study) a rat tau knock-out is unavailable. The staining seen for 0N tau in our hands was quite uniform; however, our study does not allow the discrimination of 0N3R from 0N4R. On the other hand, by Western blotting, we revealed trace amounts of 0N3R in adult mouse brain, which may in part represent the pool of proliferating neuronal progenitor cells. Overall, as the mouse brain develops, there is a transition from 0N3R (at P0) to 0N3R/0N4R at 2 weeks of age. The major isoform at 2 months of age is 0N4R, whereas the 1N4R and 2N4R bands showed much lower, comparable signal intensities. For 0N3R, however, only trace amounts were detected at this age. These findings confirm those of other tau transgenic studies in which wild-type mice were used as controls [42].

 Our study also addressed the distribution of tau in different brain areas and peripheral organs using the Tau5 antibody. Within the sensitivity of our analysis and amongst the tissues analyzed we observed detectable levels of tau only in brain. Tau5 is an antibody that if present would also detect 'big tau' that is generated by a 250-300 residue insert. An earlier study had reported tau in the rat and bovine testis; however, in this case, tau was enriched by precipitation with 35-45% ammonium sulfate [43], a method we did not use. If there is tau expression in testis, the levels must be very low. More widespread expression of tau was reported in rat tissues in another study, but the patterns of the four antibodies employed (5E2, T46, C5 and Tau1) were inconclusive and the authors indicated that the strong bands seen for retina, adrenal gland and testis were possibly due to cross-reactivity with the microtubule-associated protein MAP2C or other proteins. Importantly, these studies did not use extracts from tau knock-out mice as negative controls as we have done [27]. 

 Taken together, we demonstrate for the first time a distinct subcellular distribution of the 0N, 1N and 2N isoforms of tau in wild-type mice. The finding that 1N tau is enriched in the nucleus suggests that a deregulation of tau's nuclear functions could potentially contribute to pathological conditions. Given that murine and human tau show a 89% identity at the amino acid level, with amino-terminal splicing preserved, understanding more about the murine isoforms tau will provide important clues for understanding human tau.
